# Reading Difficulties in Parkinson’s Disease: A Stepped Care Model for Neurovisual Rehabilitation

**DOI:** 10.3233/JPD-230124

**Published:** 2023-11-03

**Authors:** Iris van der Lijn, Gera A. de Haan, Fleur E. van der Feen, Anne C.L. Vrijling, Catharina Stellingwerf, Anselm B.M. Fuermaier, Pia Langenberg, Teus van Laar, Joost Heutink

**Affiliations:** a Department of Clinical and Developmental Neuropsychology, University of Groningen, Groningen, The Netherlands; b Royal Dutch Visio, Centre of Expertise for Blind and Partially Sighted People, Huizen, The Netherlands; c Department of Neurology, University of Groningen, University Medical Centre Groningen, Groningen, The Netherlands

**Keywords:** Parkinson’s disease, reading, rehabilitation, vision disorders, visual perception, cognition, quality of life

## Abstract

**Background:**

People with Parkinson’s disease (PD) frequently experience reading difficulties. Little is known about what functional impairments distinguish people with PD with and without reading difficulties and how these should guide rehabilitation.

**Objective:**

To provide concrete advice for an efficient stepped care model for reading difficulties in PD, based on extensive functional assessments.

**Methods:**

This study included 74 people with PD in a neurovisual rehabilitation setting who underwent assessment of visual, visuoperceptual, and cognitive functions. Outcomes were compared between those with frequent (RD+; N = 55) and infrequent reading difficulties (RD–; N = 19). Aids and advice provided during rehabilitation were registered.

**Results:**

Only a few functions appeared to distinguish RD+ and RD–. Visual functions (i.e., contrast sensitivity, g = 0.76; reading acuity, g = 0.66; visual acuity, g = 0.54) and visuoperceptual functions (i.e., visual attention, g = 0.58, visual motor speed, g = 0.56) showed significant worse scores in RD+ compared to RD–. Aids and advice applied consisted mainly of optimizing refraction, improving lighting, and optimizing text size and spacing.

**Conclusion:**

The test battery showed significant differences between RD+ and RD–on only a few tests on visual and visuoperceptual functions. The applied aids and advice matched well with these impairments. Therefore, we recommend a stepped care model, starting with a short test battery on these functions. If this battery indicates functional impairments, this can be followed by standard aids and advice to improve reading. Only in case of insufficient effect additional testing should take place.

## INTRODUCTION

Parkinson’s disease (PD) is characterized by motor and many non-motor complaints, including both cognitive and visual impairments [1]. Activities that require these functions, such as participating in traffic, searching and finding objects, and reading, may become increasingly difficult [2–5].

Reading difficulty is a frequently reported complaint by people with PD [2, 5, 6]. People with PD read slower than people without PD and have difficulty with reading comprehension [7, 8]. They experience words moving [9], letters disappearing [10], blurry or double vision [11–13], and visual discomfort while reading [14]. In addition, they report difficulties with reading a text on a colored or gray background and to read better with one eye closed [10], probably to reduce binocular double vision.

Cognitive functions thought to be important for reading skills are working memory [15–17], executive functions [15, 17–19], and attention and processing speed [20–22]. Reading difficulties seem to increase with disease progression and may already be present early in the disease process [7, 23]. The association between reading difficulties and cognitive dysfunction is still contradictory in the literature, but was not studied in a large sample yet. Some studies found an association [24, 25], while others did not [8].

Several studies have reported relationships between visual impairments (like visual acuity, contrast sensitivity, and ocular motility) and reduced reading speed in PD [4, 8, 24, 26]. Also visuospatial functioning and sensitivity to crowding have been associated with reading speed in people with PD [8]. Furthermore, reduced visual processing speed and visual attention in PD may lead to reduced visual search, which seems to influence reading as well [22].

The aforementioned studies have focused on specific aspects of reading and their relationship to a particular cognitive, visual or visuoperceptual function. To the best of our knowledge, no study has examined general reading difficulties and their relationship to a wide variety of functions. Currently, rehabilitation is often preceded by extensive assessment of several functions. Apart from potentially being an unnecessary burden for people with PD, it is questionable if that is cost-effective. We need to better understand the impaired functions associated with reading difficulties in PD in order to create a lean rehabilitation process.

To manage reading difficulties, tailored neurovisual rehabilitation will be provided and evaluated. It is known that occupational therapy can improve visual functioning of people with movement disorders [27]. However, little has been reported on the availability and effectiveness of reading interventions for people with PD, beyond, for example, prism glasses and home vision therapy [28]. Hence, we will provide an overview of all reading aids and advice provided in neurovisual rehabilitation, along with how this has been received by people with PD.

This study will be the first to explore reading difficulties in people with PD and their relationship to a comprehensive set of visual, visuoperceptual and cognitive functions, using a cross-sectional design. In addition, reading aids and advice provided in neurovisual rehabilitation will be evaluated. The study aims to provide clear recommendations on an efficient stepped model for the assessment and rehabilitation of reading difficulties in people with PD.

## MATERIALS AND METHODS

### Participants and procedures

The study took place in a clinical setting of neurovisual rehabilitation, which is a combination of cognitive and low-vision rehabilitation strategies for people with a brain disorder and visual problems. This rehabilitation was provided by Royal Dutch Visio in the northern part of the Netherlands (locations Haren, Leeuwarden and Hoogeveen). All people with idiopathic PD who were referred to Royal Dutch Visio and followed a program of combined outpatient and home-based rehabilitation between August 2017 and June 2022 were eligible for the study.

During an admission interview at Royal Dutch Visio, reading difficulties were surveyed by a trained healthcare professional using a standardized questionnaire on visual complaints (see ‘Materials’). Based on the extent of the reported reading difficulties, people were allocated to a group with frequent reading difficulties (RD+) or a group with no, or infrequent reading difficulties (RD–). Table 1 shows the demographics and disease-related characteristics of both groups. Groups did not differ significantly on any of the characteristics.

**Table 1:** Demographics and disease-related characteristics of people with PD with frequent (RD+) and infrequent (RD–) reading difficulties

	Total	RD+	RD–	*p* ^a^	ES^b^
N	74	55	19
Age (y; M±SD (range))	72.0±7.7	72.8±8.5	70.0±4.6	0.08	–0.48
	(45–87)	(45–87)	(61–78)
Sex (female; *n*, %)	19, 25.7%	16, 29.1%	3, 15.8%	0.36	0.13
Education^c^ (*n*, %)				0.99	0.02
Low	11, 14,9%	8, 14.5%	3, 15.8%
Medium	25, 33.8%	18, 32.7%	7, 36.8%
High	34, 46.0%	25, 45.5%	9, 47.4%
Missing	4, 5.4%	4, 7.3%	0, 0.0%
Disease duration (y; M±SD)	9.0±6.4	8.5±6.0	10.2±7.4	0.49	–0.09
Missing (*n*, %)	9, 12.2%	8, 14.6%	1, 5.26%
H&Y stage (*n*, %)				0.27	0.25
1	3, 4.1%	1, 1.8%	2, 10.5%
2	31, 41.9%	21, 38.2%	10, 52.6%
3	24, 32.4%	19, 34.5%	5, 26.3%
≥4	8, 10.8%	7, 12.7%	1, 5.3%
Missing	8, 10.8%	7, 12.7%	1, 5.3%
LEDD^d^ (mg; M±SD)	1085.3±666.9	1065.3±699.2	1150.9±566.1	0.52	–0.08
Missing (*n*, %)	14, 18.9%	9, 16.4%	5, 26.3%
Severe neurological condition (*n*, %)	12, 16.2%	9, 16.4% ^e^	3, 15.8% ^f^	>0.99	0.01
Severe psychiatric condition (*n*, %)	0, 0.0%	0, 0.0%	0, 0.0%	–	–
Ophthalmological condition (*n*, %)^g^	37, 50.0%	29, 52.7%	8, 42.1%	0.43	0.09

ES, effect size; H&Y, Hoehn and Yahr staging [36]; LEDD, Levodopa equivalent daily dose; M, mean; mg, milligram; n, number; PD, Parkinson’s disease; RD+, people with frequent reading difficulties (often/always); RD–, people with infrequent reading difficulties (never/hardly/sometimes); SD, standard deviation. ^a^Group differences were examined by *t*-test (age), Fisher’s Exact Test (sex and severe neurological conditions), Chi-Square test (education and ophthalmological condition), Mann-Whitney U test (disease duration and LEDD), and Fishers-Freeman-Halton Exact test (H&Y stage). ^b^ES were calculated by Hedges’ g (age), Phi (sex, severe neurological condition and ophthalmological condition), Cramer’s V (education and H&Y stage), and Cohens’ d (disease duration and LEDD). ^c^Categorization based on the International Standard Classification of Education (ISCED) [51]. ^d^LEDD calculated according to protocol of Tomlinson et al. (2010) [52]. ^e^Transient ischemic attack (*n* = 1), cerebrovascular accident (*n* = 3), PD dementia (*n* = 2), thalamotomy (*n* = 1), Lewy body dementia (*n* = 2). ^f^PD dementia (*n* = 2), dementia syndrome (*n* = 1). ^g^See Supplementary Table 1.

Following the admission interview, people in both groups underwent a series of assessments in the following order: visual, visuoperceptual, and cognitive function assessments. These assessments were carried out according to pre-established protocols following the original test manuals, unless otherwise stated. Assessments were performed by trained healthcare professionals (i.e., an ophthalmologist, orthoptist, and neuropsychologist) and took approximately four hours in total. Based on the results of these assessments, a multidisciplinary team including an ophthalmologist, orthoptist, neuropsychologist, and occupational therapist gave advice, aids, and training for each individual. An occupational therapist introduced the aids and advice to the patient. At the end of the rehabilitation program, the occupational therapist evaluated with the patient which aids and advice had contributed to reducing visual complaints. Only aids and advice aimed at improving reading were analyzed in this study. Patients were on their regular medication dosages during the assessments and rehabilitation trajectory.

Data were collected from people who had given written consent for the collection and use of pseudonymized data from their medical records for the purpose of this study. Consent could be withdrawn at any time. The decision to consent or not did not affect the care provided. The Medical Research Ethics Committees of the University Medical Center Groningen deemed that this study did not fall within the scope of the Dutch Medical Research Involving Human Subjects Act (WMO), since all data were collected in the context of standard care.

## MATERIALS

### Self-reported reading difficulties

The Cerebral Visual Complaints questionnaire (CVCq) is a 43-item questionnaire extended on the original Cerebral Vision Screening questionnaire developed by Kerkhoff, Schaub, and Zihl [29], and supplemented with questions from the Screening Visual Complaints questionnaire [30, 31]. For the analysis of this study, only questions assessing reading difficulties were included. The first question determined the presence of reading difficulties (i.e., “Do you have trouble reading due to your eyesight?”; Likert scale ‘never/hardly’, ‘sometimes‘, or ‘often/always‘). In case reading difficulties were present (‘sometimes‘ or ‘often/always‘), the nature of reading difficulties was assessed using the second semi-structured question, by which people could indicate one or multiple pre-described response options (i.e., trouble staying on the same line, experiencing dancing letters, skipping words, trouble finding the beginning of a line) as well as describe the reading difficulty themselves (open-ended part of the question). In addition, people were asked about the influence of light during visual activities, including reading.

### Function assessments and data collected from medical records

#### Assessment of visual, visuoperceptual, and cognitive functions

The tests comprising the assessments of visual, visuoperceptual, and cognitive functions are presented in Table 2 and classified according to earlier presented methodology [32]. Visual functions were determined using both eyes (binocular), except for color vision and eye motility, which were determined separately for each eye (monocular). A specialized orthoptist evaluated the reliability of visual field measurements. Unreliable results were excluded from the analyses (Esterman: ≥10% false positives or ≥20% fixation losses; Goldmann: insufficient central fixation or non-reproducible response). To ensure subtle color vision deficits were not missed, the Lanthony test [33] for color vision was used in case no deficit was apparent on the Farnsworth test [34]. Smooth pursuit was assessed by asking people to follow a moving light horizontally and vertically, to approximately 40 degrees from the center. The light was moved in eight different directions to assess eye motility (six cardinal directions and upward and downward). Saccades were assessed by asking people to look alternately from one object to another, approximately 40 cm apart, horizontally and vertically.

**Table 2:** Assessment of visual, visuoperceptual, and cognitive functions: tests and classification of impairment

Function	Test	Impaired^a^
**Visual functions**
Visual acuity	ETDRS [53] (500 lux, 4 m)	<0.8 Snellen (Logmar <0.1)
Contrast sensitivity	Vistech [54], Gecko [55] (500 lux, 3 m)	Peak log contrast sensitivity <1.40
Reading acuity (near-distance visual acuity)	LEO reading chart: reading distance (m)/print size (M-unit) [56]	<visual acuity/2
Visual field	Goldmann, Esterman	Presence of binocular absolute scotoma
Color vision	Farnsworth D-15 [34], Lanthony D-15 [33] (400 lux)	Impaired
Stereopsis	Lang [57]/TNO [58]/House Fly [59]	Stereopsis absent
Pupillary light reflex	Swinging light test (10 and 500 lux)	Impaired
Eye alignment	Cover/uncover test (30 cm)	Not aligned, tropias
Eye motility	Orthoptist assessment	Impaired in one or both eyes
Saccades	Orthoptist assessment	Impaired horizontally and/or vertically
Smooth pursuit	Orthoptist assessment	Impaired horizontally and/or vertically
Convergence	Orthoptist assessment	>10 cm
Nystagmus	Orthoptist assessment	Nystagmus present
Blink rate	Orthoptist assessment	Low
Optokinetic nystagmus	Orthoptist assessment	Impaired horizontally and/or vertically
Vestibulo-ocular reflex	Orthoptist assessment	Impaired horizontally and/or vertically
**Visuoperceptual functions[35]**
Figure ground segmentation	L-POST Figure ground segmentation [60]	<17th percentile
Shape ratio	L-POST Shape ratio [60]	<17th percentile
Motion detection	L-POST Motion detection [60]	<17th percentile
Visual motor speed	Trail making Test A [61]	<17th percentile
Visual motor speed in a task with high mental effort	Trail making Test B [61]	<17th percentile
Mental flexibility	Trail making Test B/A [61]	<17th percentile
Visual attention/spatial cognition/crowding	Bells Test [62]	<17th percentile
Visual constructive skills	Taylor Complex Figure [63]	<17th percentile
Visual search/grouping	Dot Counting Task [64]	<10th percentile
Visual load/crowding	Crowding Task [35]	<15th percentile
Simultanagnosia	Birthday Party Test [65]	<17th percentile
Visuospatial memory	Corsi Block Tapping Task [66]	<17th percentile
Object perception	Silhouettes [64]	<17th percentile
**Cognitive functions**
Short term memory (span capacity), focused/sustained attention	Digit Span-forward [67,68]	<14–19th percentile
Working memory, focused/sustained attention	Digit Span-backward [67,68]	<14–19th percentile
Working memory, focused/sustained attention	Digit Span-sorting [67,68]	<14–19th percentile
Short term/working memory, focused/sustained attention	Digit Span-total [67,68]	<14–19th percentile
Verbal memory –encoding	15 Words Test [69]	<17th percentile
Verbal memory –retention	15 Words Test-recall [69]	<17th percentile
Verbal fluency, executive functioning	Letter Fluency [70]	<17th percentile
Anxiety symptoms	HADS Anxiety [71]	Raw score >11
Depression symptoms	HADS Depression [71]	Raw score >11

ETDRS, Early Treatment Diabetic Retinopathy Study; HADS: Hospital Anxiety Depression Scale; LEO, Laboratory of Experimental Ophthalmology; L-POST, Leuven Perceptual Organization Screening Test. ^a^We chose to define impairment as mild decline in function to avoid overlooking mild impairment.

Visuoperceptual functions were assessed using the DiaNAH-battery [35] on a 24” Wacom tablet, programmed by Metrisquare Diagnosis (http://www.diagnosis.com). The cognitive tests measured cognition without a visual component and were performed in the following order: letter fluency, digit span, 15 words test. These tests were conducted verbally, with the neuropsychologist writing down the answers given. The analysis of anxiety and depressive symptoms (using the self-report questionnaire Hospital Anxiety and Depression Scale (HADS)) was also part of the cognitive battery. Scores on each test were calculated following the original manuals.

#### Data from medical files

Disease-related data were obtained from the medical records of each individual, provided upon referral by their treating neurologist and/or ophthalmologist. When a person had not previously been seen by an ophthalmologist, an ophthalmological examination was performed at Royal Dutch Visio. Data obtained included disease duration, Hoehn and Yahr stage (H&Y) [36], current medication, and neurological, ophthalmological and psychiatric comorbidities. In addition, applied aids and advice to improve reading, as well as its ability to effectively reduce reading difficulties according to patient reports, were obtained from the documentation of the occupational therapist at Royal Dutch Visio.

### Data analysis

All analyses were performed with SPSS 27 [37].

#### Reading difficulties

The semi-structured question of the CVCq was analyzed if people had indicated reading difficulties (‘sometimes‘ or ‘often/always’). Responses to the open-ended part of the question were categorized based on similarities in content by two authors (IvdL & PL). Subsequently, the frequencies of both the structured response options and these categories were calculated.

#### Reading difficulties and the relationship with visual, visuoperceptual and cognitive functions

Mean function scores were compared between the RD+ (reading difficulties ‘often/always’) and RD–groups (reading difficulties ‘never/hardly’ or ‘sometimes’). These scores were mostly calculated from raw scores. Functions like visual field, the presence of stereopsis, eye alignment, convergence, nystagmus, and blink rate were scored as either 0 (not impaired) or 1 (impaired). Color vision, pupillary light reflex and eye motility were scored 0 (no impairment), 1 (impaired in one eye) or 2 (impaired in both eyes). Saccades, smooth pursuit, optokinetic nystagmus, and vestibulo-ocular reflex were scored 0 (no impairment), 1 (impaired either horizontally or vertically) or 2 (impaired in both. directions).

Hedges’ g was used as an effect size (ES) for the difference in mean function scores between the groups, which is a less biased alternative to Cohen’s d in case of small and different sample sizes [38]. ES were complemented by the corresponding 95% confidence interval (CI). In addition, we calculated the percentage of people with an impaired function score per group, as described in Table 2.

To detect the relationship between visual, visuoperceptual, and cognitive functions and reading difficulties, the overall weighted mean ES was calculated for each of these three categories. The weight of each individual ES on the overall ES was based on sample sizes of each group, taking missing values into account (see SupplementaryTable 2). Each individual ES was multiplied by the number of present values (sample size minus number of missing values) and hereafter all ES per category were summed and divided by the total sample. size.

#### Handling missing data

Reasons for missing data were related to time constraints, covid-19 constraints, physical constraints, fatigue, unreliability of test results, and the judgement of the multidisciplinary team that additional assessments were not necessary to compensate the reading difficulty. The amount of missing data per variable is presented in Supplementary Table 2. To maximize sample size for individual ES calculations, pairwise deletion of missing values was applied.

#### Reading aids and advice

Aids and advice provided during neurovisual rehabilitation were categorized and the frequency of use and effectiveness of each advice or aid was calculated. An aid or advice was found to be effective if the patient indicated that it sufficiently alleviated reading difficulties.

## RESULTS

A total of 83 people was referred to Royal Dutch Visio and completed the standard neurovisual rehabilitation trajectory. Three participants (3.6%) proved not to have idiopathic PD, but were classified as atypical parkinsonism, and therefore excluded from this study. Six others (7.2%) did not consent the collection and use of their pseudonymized data. The remaining sample consisted of 74 eligible people with PD (see Table 1).

### Reading difficulties

Of the 74 eligible people with PD, 65 (87.8%) indicated to have reading difficulties on the CVCq (10 (13.5%) indicated ‘sometimes’, 55 (74.3%) ‘often/always’) and nine (12.2%) did not (they indicated ‘never/hardly’). Out of the 65 people with reading difficulties, all except one (for reasons unknown) responded to the semi-closed CVCq question. Individuals reported to experience a variety of difficulties during reading, which are presented in Table 3.

**Table 3:** Reported difficulties experienced during reading by people with PD with reading difficulties (‘sometimes’ or ‘often/always’)

Reported problem during reading (*n* = 64)	N	%
**Pre-described response options**
Trouble staying on the same line	23	35.9%
Dancing letters	22	34.4%
Skip words	22	34.4%
Trouble finding the beginning of a line	18	28.1%
**Open-ended part of question**
Fatiguing/Tiring/strenuous	21	32.8%
Double vision	14	21.9%
Unclear vision/trouble focusing	14	21.9%
Letters are too small	12	18.8%
Problems with glasses	3	4.7%
Moving sentences/words	2	3.1%
Needing more time/subtitles go too fast	2	3.1%
Other	10	15.6%
Not visual/cognition (i.e., problems with attention/concentration)	7	10.9%

People could describe more than one problem; ‘Other’ difficulties were: “painful eyes”, “I read bit by bit; in pieces”, “small membranes for my eyes”, “watery eyes”, “d and b are hard to distinguish”, “unstable image”, “sometimes I linger in one place, and read again”, “don’t get full words in view”, “there is a shadow behind the letters”, “constricted view"

The different reading difficulties predescribed by the CVCq were reported with about the same frequency. About one-third of the people reported dancing letters (34.4%), skipping words (34.4%), having trouble finding the beginning of the sentence (28.1%) or having problems to stay on the same line while reading (35.9%). Several difficulties were also spontaneously reported quite frequently, such as becoming fatigued during reading (32.8%), experiencing double or unclear vision (21.9%), having trouble focusing (21.9%), or experiencing letters as too small (18.8%).

People were also asked about the influence of light on their vision. In total 46 people (62.2%) indicated an increased need for light, of which 38 (83.0%) indicated to need extra light when reading. Thirty-seven people (50.0%) indicated increased light sensitivity (discomfort glare), of which four (10.5%) indicated this while reading.

### Reading difficulties and the relationship to visual, visuoperceptual, and cognitive functions

Table 4 presents the functional differences between the RD+ and RD–groups. The RD+ group scored especially lower than the RD–group on contrast sensitivity (g = 0.76, 95% CI [0.19, 1.33]), reading acuity (g = 0.66, 95% CI [0.11, 1.22]), and visual acuity (g = 0.54, 95% CI [0.01, 1.07]), as well as on visual attention/spatial cognition/crowding (Bells Test; g = 0.58, 95% CI [0.05, 1.11]) and visual motor speed (Trail Making Test B; g = 0.56, 95% CI [–0.01, 1.12]). CI-bounds were broad, but did not cross zero for these variables (except for the Trail Making Test B), and are therefore significant (α< 0.05).

**Table 4:** Differences in visual, visuoperceptual, and cognitive functions between people with PD with frequent (RD+) vs. infrequent (RD–) reading difficulties

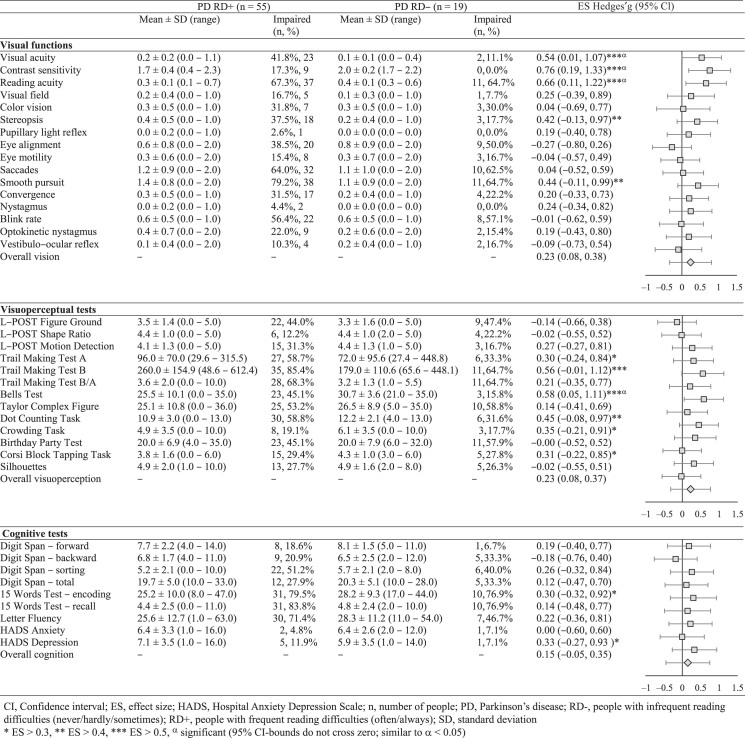

The overall weighted mean ES of each category were all small, but the largest differences between RD+ and RD–groups were shown for visual functions (g = 0.24, 95% CI [–0.33, 0.80]) and visuoperceptual functions (g = 0.23, 95% CI [–0.31, 0.76]). A smaller overall ES was found for cognitive functions (g = 0.15, 95% CI [–0.44, 0.74]). These ES show that people in the RD+ group attained relatively lower functional scores than people in the RD–group, although CI-bounds are broad and cross zero. The RD+ group attained lower scores than the RD–group on 29 of 38 tests.

Percentages of impairment showed that impairments were present in both groups (see Table 4). Consistent with the ES, these percentages show that more impairments are present in the RD+ group compared to the RD–group. Three functions were impaired exclusively in the RD+ group, being contrast sensitivity, pupillary light reflex, and nystagmus. The latter two were also uncommon in the RD+ group (2.6% and 4.4%, respectively) and showed small ES. Contrarily, contrast sensitivity was impaired in 17.3% of the RD+ group and showed the largest ES.

### Reading aids and advice

Sixty people, from both the RD+ (N = 47) and RD–(N = 13) groups, received aids and advice for reading difficulties. Of the RD+ group, eight people did not enter the rehabilitation trajectory to receive aids and advice. Reasons for this were deteriorating health, relocation, or referral to an ophthalmologist because of a present ophthalmological condition. Of the RD–group, six did not receive aids and advice for lack of difficulties.

Aids and advice that were most frequently provided were the use of glasses, magnification, task lighting, e-reader/tablet/computer, reduction of visual input and fostering focus by covering surrounding text, and auditory alternatives (see Table 5).

**Table 5:** Aids and advice for reading improvement provided in neurovisual rehabilitation

	Advice	Aid	Effective/Total (n/n)
Glasses	Use better correction	(New) glasses	14/14
	Use prism correction	Prism glasses	5/9
	Do not use multifocal glasses	Separate reading glasses	9/15
Magnification	Magnification	–	26/28
	Magnification	Magnifying lamp	6/8
	Magnification	Screen/electronic magnifier	4/5
	Magnification	Magnifying glasses	1/1
	Magnification	Magnifying glass	0/1
Lighting	Increase task lighting	Reading/task lamp	28/32
	Reduce discomfort glare	Sunshade/awning	1/1
Electronic optimization of text formatting	Optimize text formatting	E-reader/Tablet/Computer	24/30
	Increase line spacing	E-reader/Tablet/Computer	8/10
	Increasing contrast	E-reader/Tablet/Computer	6/6
	Bold printing of letters	E-reader/Tablet/Computer	1/1
	Increase brightness	E-reader/Tablet/Computer	1/1
	Increase resolution	E-reader/Tablet/Computer	1/1
	Zoom in	E-reader/Tablet/Computer	0/1
Reduction of distracting visual input/Foster focus	Cover surrounding text	–	7/11
	Cover surrounding text	Reading overlay (below text)	14/19
	Cover surrounding text	Reading frame (around text)	5/6
	Use aid for finding and staying on the same line	Reading ruler	6/6
Reduction of distortive input from one eye (e.g., deviant ocular alignment/double vision)	Completely cover one eye	Eye patch	12/18
Partially cover one eye	Monocular filter glasses	2/3
Posture/Text positioning	Optimize seating posture	–	9/9
	Optimize seating posture/text position	Reading stand	11/14
	Optimize seating posture/text position	Laptop stand	1/1
	Decrease reading distance	–	7/7
	Increase reading distance	–	1/1
	Do not hold text, but put down	–	2/2
	Do not lay text flat but hold it up	–	1/1
	Move text in time	–	1/1
Schedule	Taking breaks (reading briefly several times a day rather than once for a longer period)	–	4/4
	Reading at a particular time of the day	–	0/1
Auditory alternatives	Spoken books	Daisy player	18/26
	Spoken subtitles	Go Box	14/16
	Text-to-speech	E-reader/Tablet/Computer	3/3
	Spoken alternative (e.g., news)	Radio	2/2
	Record memos instead of writing them down	Memorecorder	1/1
Other	Increase blink frequency (conscious blinking)	–	5/5
	Read slower	–	1/1
	Reading with heavy object to reduce tremor	–	1/1
	Placing glasses in a fixed predetermined place	–	1/1

n, number of people.

Most people experienced the aids and advice to be effective in reducing reading difficulties. Prism glasses had variable effects, with some people still reporting double vision, which is due to the highly variable double vision. Separate reading glasses were mostly effective in alleviating reading difficulties. Some people had motor problems with changing glasses. The same holds true for some people using e-readers, tablets or computers. Operating these devices was difficult to learn and required separate training. Sometimes people preferred not to use auditory alternatives because they did not want to give up. reading.

## DISCUSSION

This is the first study that explored reading difficulties in people with PD and their relationship to an extensive set of visual, visuoperceptual, and cognitive functions. The goal of this study was to identify which functions are important to assess in order to design care as efficiently as possible, and to provide an overview of effective aids and advice for reading improvement.

People reported dancing letters, skipping words, having trouble staying on the same line or finding the beginning of a new line, as well as fatigue or unclear or double vision. These findings are similar to those reported in previous studies [9, 11–13].

The presence of these reading difficulties was most closely related to contrast sensitivity, reading acuity (near-distance visual acuity), visual acuity, visual attention/spatial cognition/crowding (Bells test), and visual motor speed (Trail Making Test B).

Our findings are not surprising, as visual and visuoperceptual functions were previously found to be related to reading difficulties [39, 40]. Visual acuity and reading acuity are important for reading performance, e.g., for letter and word identification [39, 41]. Spatial attention is necessary for the decoding of written information and determines word recognition and comprehension [39, 42]. As reading follows a precise direction, it requires an adequate development of visual attention as well [43]. Intact mental flexibility and inhibition ensures readers to shift between text meaning, letter-sound information, and syntactic information, and to prevent the activation of incorrect meaning or irrelevant connections [44]. Visual-motor speed includes the ability to determine and maintain line orientation [45]. Reduced visual-motor speed may therefore be another explanation for difficulty following a line and finding the beginning of a new line reported by people with. PD.

Impairments in contrast sensitivity were the best discriminator between both groups, and these impairments were exclusively present in people with frequent reading difficulties. Previous data confirm the importance of contrast sensitivity, even exceeding the importance of visual acuity [46]. It is plausible that a reduced contrast sensitivity partly explains the reported problems in this study. People may experience more problems when letters are less easy to identify (e.g., having word finding difficulties, difficulties finding the right line, or experience unclear vision, which may cause the fatigue associated with reading) [47, 48].

However, most functional impairments occurred to a similar extent in both groups. This is surprising for functions like oculomotor impairments and crowding, because these were related to unclear or double vision, dancing letters, and getting distracted by irrelevant visual information in previous studies [8, 39, 49]. Cognitive dysfunction was not related to reading difficulties and is therefore unlikely to play a role in reading difficulties. Although people did attribute their reading difficulty partly on their inability to concentrate in our and a previous study [3], this finding is consistent with previous literature indicating that reading difficulties can exist without cognitive impairment [7].

At group level, the use and effectiveness of the most commonly provided aids and advices fitted quite well with the most important impairments in people with PD and reading difficulties.

### Clinical implications

Reading difficulties are among the most prevalent visual complaints in people with PD, with a substantial impact on their daily lives [2, 6]. Therefore, it is important to ask about these difficulties in clinical practice. In case of their presence, an assessment of visual and visuoperceptual functions may provide additional insight into impairments that contribute to the development and persistence of reading difficulties in people with PD, which may guide individually tailored rehabilitation (e.g., new glasses with optimal refraction or text magnification in case of impaired visual acuity, or providing optimal contrast by the use of a display in case of impaired contrast sensitivity). As cognitive dysfunction was not related to reading difficulties, non-visual cognitive testing need not to be an essential part of the assessment to evaluate reading difficulties.

Based on our results we suggest not to administer an extensive test battery as we did in our study, but to use a stepped care model. Only a limited number of functions seemed to be related to reading difficulties. We therefore advise, as a first step, to start assessing these few specific functions in the case of reading difficulties (i.e., contrast sensitivity, reading acuity, and visual acuity, and the visuoperceptual functions visual attention/spatial cognition/crowding (Bells test), and visual motor speed (Trail Making Test B)), because these seem to play an important role in reading difficulties. These assessments would take less than half an hour. In case of impairments in one of these functions, aids and advice specifically targeting these dysfunctions may be most helpful. If these measures show insufficient effect, additional testing might take place.

### Strengths, limitations, and recommendations for future research

This study was the first to explore the relationship between reading difficulties and an extensive set of visual, visuoperceptual and cognitive functions in people with PD. It is also the first study that provides an overview of reading interventions that were applied in neurovisual rehabilitation. The sample size of the group without reading difficulties was limited, especially due to the presence of missing values. Therefore, future research should examine larger samples, making the study less exploratory and giving it more power to draw stronger. conclusions.

The differences between both groups might have been underestimated in our study, as the people with no or infrequent reading difficulties (‘never/hardly’ and ‘sometimes’) were all put together in one group. Differences between the groups are expected to be more apparent when the comparison group would have had no reading difficulties at all. Moreover, our method for group allocation may lack in robustness, as it relied solely on a single variable (question of the CVCq). To improve group allocation, future studies could implement a more sophisticated approach that considers multiple variables.

Although demographic and disease-related characteristics did not significantly differ between the groups, age and disease severity may be confounding factors, with medium and large ES, respectively. We did not correct for these variables in the analyses. As having a higher age and disease stage may be part of the risk factors for having reading difficulties, controlling for this may partially even out the presence of reading difficulties, making it harder to draw conclusions on other relating factors. Whether age and disease severity are actually related to reading difficulties could be subject of future research. Earlier studies did show that age and disease severity are positively related to the presence of visual and cognitive symptoms [6, 50].

### Conclusion

This exploratory cross-sectional study showed that visual and visuoperceptual impairment play an important role in the experience of reading difficulties in people with PD. Especially contrast sensitivity, reading acuity, visual acuity, visual attention, and visual motor speed seem to distinguish people with frequent and people with no or infrequent reading difficulties. Reading aids and advice currently applied in neurovisual rehabilitation seem to fit nicely with the impairments that are most commonly present in people with PD with reading difficulties. Therefore, we suggest a stepped care model in which the test battery is minimized and rehabilitation is focused on these specific impairments. Additional testing should only take place in case of insufficient effect. This would be an efficient and cost-effective approach to rehabilitation of reading difficulties in people with PD.

## Supplementary Material

Supplementary Material

## Data Availability

Data that support the findings of this study are available for verification purposes upon reasonable request for 10 years after publication of the article. Requests can be submitted via j.h.c.heutink@rug.nl. Data are not available for reuse because Royal Dutch Visio is the rights holder of the data, and due to privacy and ethical restrictions (i.e., the combination of variables can potentially lead to participants being identified).
