# Supplementary material for: Reading Difficulties in Parkinson’s Disease: A Stepped Care Model for Neurovisual Rehabilitation
Source: J Parkinsons Dis. 2023 Nov 3;13(7):1225–37. doi: 10.3233/JPD-230124 (PMC10657659; doi:10.3233/JPD-230124)
Supplement: Supplementary Material [file jpd-13-jpd230124-s001.pdf]

Supplementary Material

Reading Difficulties in Parkinson's Disease: A Stepped Care Model for Neurovisual Rehabilitation

Supplementary Table 1. Ophthalmological conditions in people with PD with frequent (RD+) and infrequent (RD-) reading difficulties

	Total (n = 74)		RD+ (n = 55)		RD- (n = 19)	
	N	%	N	%	N	%
Cataract	12	16.2%	9	16.4%	3	15.8%
Macular degeneration	11	14.9%	10	18.2%	1	5.3%
Dry eyes disease	4	5.4%	4	7.3%	0	0%
Retinopathy	4	5.4%	3	5.5%	1	5.3%
Glaucoma	3	4.1%	2	3.6%	1	5.3%
Macular abnormality	3	4.1%	2	3.6%	1	5.3%
Corneal abnormality (including pterygium)	3	4.1%	3	5.5%	0	0%
Eye movement disorder	3	4.1%	1	1.8%	2	10.5%
Visual field defect	2	2.7%	2	3.6%	0	0%
Cloudy vitreous humor	1	1.4%	0	0%	1	5.3%
Blindness in one eye	1	1.4%	1	1.8%	0	0%
History of melanoma in the eye	1	1.4%	1	1.8%	0	0%
Missing	4	5.4%	2	3.6%	2	10.5%

PD, Parkinson's disease; RD+, people with frequent reading difficulties (often/always); RD-, people with infrequent reading difficulties (never/hardly/sometimes)

Supplementary Table 2. Missing data of visual, visuoperceptual, and cognitive functions in people with PD with frequent (RD+) and infrequent (RD-) reading difficulties

	Total (n = 74)		RD+ (n = 55)		RD- (n = 19)	
	N	%	N	%	N	%
**Visual functions**						
Visual acuity	1	1.4%	0	0.0%	1	5.3%
Contrast sensitivity	7	8.1%	4	5.5%	3	15.8%
Reading acuity	2	2.7%	0	0.0%	2	10.5%
Visual field	31	41.9%	25	45.5%	6	31.6%
Color vision	42	56.8%	33	60.0%	9	47.4%
Stereopsis	9	12.2%	7	12.7%	2	10.5%
Pupillary light reflex	21	28.4%	17	30.9%	4	21.1%
Eye alignment	4	5.4%	3	5.5%	1	5.3%
Eye motility	4	5.4%	3	5.5%	1	5.3%
Saccades	8	10.8%	5	9.1%	3	15.8%
Smooth pursuit	9	12.2%	7	12.7%	2	10.5%
Convergence	2	2.7%	1	1.8%	1	5.3%
Nystagmus	14	18.9%	10	18.2%	4	21.1%
Blink rate	21	28.4%	16	29.1%	5	26.3%
Optokinetic nystagmus	20	27.0%	14	25.5%	6	31.6%
Vestibulo-ocular reflex	23	31.1%	16	29.1%	7	36.8%
**Visuoperceptual tests**						
L-POST Figure Ground	5	6.8%	5	9.1%	0	0.0%
L-POST Shape Ratio	7	9.5%	6	10.9%	1	5.3%
L-POST Motion Detection	8	10.8%	7	12.7%	1	5.3%
Trail Making Test A	10	13.5%	9	16.4%	1	5.3%
Trail Making Test B	16	21.6%	14	25.5%	2	10.5%
Trail Making Test B/A	16	21.6%	14	25.5%	2	10.5%
Bells Test	4	5.4%	4	7.3%	0	0.0%
Taylor Complex Figure	10	13.5%	8	14.5%	2	10.5%
Dot Counting Task	4	5.4%	4	7.3%	0	0.0%
Crowding Task	15	20.3%	13	23.6%	2	10.5%
Birthday Party Test	4	5.4%	4	7.3%	0	0.0%
Corsi Block Tapping Task	5	6.8%	4	7.3%	1	5.3%
Silhouettes	8	10.8%	8	14.5%	0	0.0%
**Cognitive tests**						
Digit Span - forward	16	21.6%	12	21.8%	4	21.1%
Digit Span - backward	16	21.6%	12	21.8%	4	21.1%
Digit Span - sorting	16	21.6%	12	21.8%	4	21.1%
Digit Span - total	20	27.0%	16	29.1%	4	21.1%
15 Words Test - encoding	22	29.7%	16	29.1%	6	31.6%
15 Words Test - recall	24	32.4%	18	32.7%	6	31.6%
Letter Fluency	17	23.0%	13	23.6%	4	21.1%
HADS Anxiety	18	24.3%	13	23.6%	5	26.3%
HADS Depression	18	24.3%	13	23.6%	5	26.3%

PD, Parkinson's disease; RD+, people with frequent reading difficulties (often/always); RD-, people with infrequent reading difficulties (never/hardly/sometimes)
